# Circular RNA circCD44 targets methyltransferase‐like 3/homeobox containing 1 to facilitate esophageal squamous cell carcinoma progression

**DOI:** 10.1002/kjm2.12950

**Published:** 2025-02-26

**Authors:** Wei‐Wen Zou, Yan‐Fang Liao, Xi‐Rui Huang, Wen‐Song Lin, Shan‐Ming Lu

**Affiliations:** ^1^ Department of Pathology Longgang Center Hospital of Shenzhen Shenzhen Guangdong PR China

**Keywords:** circCD44, esophageal squamous cell carcinoma, HMBOX1, m^6^A, METTL3

## Abstract

Esophageal squamous cell carcinoma (ESCC) is a prevalent gastrointestinal malignancy with unclear functional mechanisms of circular RNAs. This study focused on investigating the role of circCD44 in ESCC progression. The expression of circCD44 and homeobox containing 1 (HMBOX1) was found to be elevated in ESCC cell lines. The overexpression of circCD44 or HMBOX1 enhanced proliferation and invasion, while inhibiting apoptosis in ESCC cells, whereas knockdown of these genes had inverse effects. METTL3 was observed to bind to circCD44 and HMBOX1 mRNA, promoting m^6^A modification in HMBOX1 mRNA and subsequent HMBOX1 protein expression. Knockdown of METTL3 or HMBOX1 attenuated the oncogenic effects of circCD44 overexpression both in vitro and in vivo. These findings suggest that circCD44 promotes ESCC progression by facilitating HMBOX1 mRNA m^6^A modification and protein expression through METTL3 binding, providing insights into the molecular mechanisms underlying ESCC pathogenesis.

## 1 INTRODUCTION

Esophageal cancer ranks 11th among the most commonly diagnosed cancers worldwide, with the highest incidence rates in Eastern Asia and Eastern Africa.^1^ Esophageal squamous cell carcinoma (ESCC) is the most prevalent histological type of esophageal cancer and is believed to develop from esophageal squamous cell dysplasia.^2^ Low socioeconomic status and tobacco/alcohol consumption constitute major causative factors in ESCC.^3^ Despite recent advances in treatment strategies and the emergence of new therapeutic approaches, the prognosis for advanced ESCC remains poor and needs further improvement.^4^ Molecular interactions underlying ESCC pathogenesis are currently being investigated to identify appropriate therapeutic agents and improve prognoses.^5^

Circular RNAs (circRNAs), a class of single‐stranded covalently closed RNAs, have been recognized as important regulatory molecules in cancers.^6^ Many circRNAs, such as circCYP24A1, circPUM1, and circFNDC3B, have been shown to regulate cancer hallmarks in ESCC.^7^, ^8^, ^9^ CircCD44, a newly identified circRNA, is aberrantly expressed in cancers including triple‐negative breast cancer, glioblastoma multiforme, and colorectal cancer.^10^, ^11^, ^12^ Moreover, circCD44 can potentiate the malignancy of ESCC cells by sponging microRNA (miR)‐23b‐5p to activate the TAB1/NF‐κB pathway.^13^ Apart from acting as miR sponges, circRNAs can also interact with RNA‐binding proteins (RBPs), thereby affecting the fate of target mRNAs of these RBPs.^14^ Therefore, circCD44 may regulate ESCC progression via other targets, the discovery of which may support the future clinical utilization of circCD44 as a biomarker or target for ESCC.

Homeobox containing 1 (HMBOX1), associated with telomeric DNA, exhibits high expression in squamous cell carcinoma of the lung and correlates with tumor growth.^15^ This suggests that HMBOX1 may also play a role in squamous cell carcinoma of the esophagus. HMBOX1 mRNA is highly methylated at N^6^‐adenosine (m^6^A) in cancer cells, resulting in chromosome abnormalities and aggressive phenotypes.^16^ Intriguingly, the m^6^A methyltransferase METTL3 regulates the aggressiveness of ESCC.^17^, ^18^ Therefore, METTL3 may exert its effect on ESCC through m^6^A methylation of HMBOX1 mRNA. Considering METTL3 as an RBP, we searched the starBase database for circCD44‐METTL3 interactions and found METTL3‐binding sites in circCD44. This suggests that circCD44 may regulate ESCC progression through METTL3‐dependent m^6^A methylation of HMBOX1 mRNA. Based on the above, this study hypothesized that circCD44 promotes ESCC development by regulating the METTL3/HMBOX1 axis, uncovering novel molecular mechanisms underlying the pathogenesis of ESCC.

## 2 MATERIALS AND METHODS

### 2.1 Cell culture and transfection

Human ESCC cell lines TE‐1 and KYSE30 and human esophageal epithelial Het‐1A cells (Shanghai Cell Bank of the Chinese Academy of Sciences) were cultured in RPMI 1640 medium (11875093, Gibco, New York, USA) plus 10% FBS (16140071, Gibco) at 37°C with 5% CO_2_. Overexpression vectors (OE‐circCD44, OE‐METTL3, and OE‐HMBOX1), knockdown vectors (si‐circCD44, si‐METTL3, and si‐HMBOX1), and negative controls (OE‐NC and si‐NC) (all from GenePharma, Shanghai, China) were transfected into ESCC cells pre‐seeded in six‐well plates using Lipofectamine 3000 (L3000015, Invitrogen, New York, USA). Transfection effectiveness was evaluated using RT‐qPCR and western blotting 48 h after transfection.

### 2.2 CCK‐8 assay

For viability assessment, transfected cells were cultured in a 96‐well plate for 72 h and then treated with 10 μL of CCK‐8 solution (C0037, Beyotime, Shanghai, China) for 4 h. The absorbance at 450 nm was measured using a microplate reader (Bio‐Rad, Hercules, CA, USA).

### 2.3 Transwell assay

Matrigel‐precoated Transwell inserts (Corning, NY, USA) were used to assess cell invasion. Transfected cells were transferred to the upper compartments (4 × 10^4^ cells/well), while the lower compartments were loaded with 10% FBS. After 48 h, invasive cells were stained with crystal violet and then counted under a microscope at 100× magnification.

### 2.4 Flow cytometry

After digestion, 1 × 10^6^ cells were washed twice with pre‐cooled PBS and resuspended in 100 μL of 1× binding buffer. The cells were incubated with Annexin V‐FITC and PI (5 μL each, BMS500FI‐300, eBioscience) at room temperature for 10 min in the dark and then resuspended in 400 μL of 1× binding buffer. The sample was analyzed by flow cytometry within 1 h to detect apoptosis.

### 2.5 Subcutaneous tumor transplantation in nude mice

TE‐1 cells in the logarithmic growth phase after transfection were harvested at around 80%–90% confluency; the culture medium was refreshed the night before the cells were collected. After trypsin digestion, the cells were washed twice with pre‐cooled PBS and resuspended in serum‐free medium to a concentration of 5 × 10^7^ cells/mL. Six‐week‐old nude mice, weighing about 18–20 g (five mice per group), were implanted with 0.1 mL of the TE‐1 cell suspension in the middle and upper part of their groin. The experiment was terminated when the tumor size of the control group reached 1 cm^3^. The tumors were isolated, and their size and weight were measured.

### 2.6 RNA immunoprecipitation

RNA immunoprecipitation (RIP) was used to detect the binding of METTL3 protein to circCD44 and HMBOX1 mRNA. Cells were lysed with RIPA buffer in an ice bath. Part of the cell extract was used as the input, and another part was incubated with antibodies. Briefly, 50 μL of magnetic beads were suspended in 100 μL of RIP wash buffer and incubated with 5 μL of rabbit anti‐human METTL3 antibodies (1:1000, ab195352, Abcam, Cambridge, UK) or goat anti‐human IgG antibodies for 30 min at room temperature. The bead–antibody complexes were suspended in 900 μL of RIP wash buffer and incubated with 100 μL of the cell extract at 4°C. The bead–protein complexes were collected using a magnetic rack. The sample and input were treated with proteinase K to isolate RNA for RT‐qPCR to detect the expression of circCD44 and HMBOX1 mRNA.

### 2.7 Me‐RIP

Extracted RNA was mixed with 450 μL of IP buffer and 2 μL of RNase inhibitor and then treated in an automatic ultrasonic disruptor; 50 μL of the RNA sample was stored at −80°C as an input, and the remaining 400 μL was assigned to the IP group, to which 20 μL of IP buffer and 4 μg of antibodies (m^6^A or IgG) were added. After magnetic bead adsorption, the IP and input samples were subjected to phenol–chloroform RNA extraction, followed by RT‐qPCR detection of HMBOX1 mRNA.

### 2.8 RT‐qPCR

One microgram of total RNA, extracted using TRIzol reagent (15596018, Invitrogen), was synthesized into cDNA using a PrimeScript RT reagent kit (3733, TaKaRa, Japan). qPCR was performed using a SYBR Green qPCR master mix (330500, Qiagen, Germany) and a Bio‐Rad CFX384 Touch detection system. All reactions were performed in triplicate. The data presented are the averages of 2^−ΔΔCt^ from at least three independent experiments, normalized to GAPDH. The primers used for amplification are listed in Table 1.

**TABLE 1:** Primer sequences used in the study.

Primers	Sequences (5′–3′)
CircCD44‐F	CCAGAGGACAGTTCCTGGAC
CircCD44‐R	CGATATCCCTCATGCCATCT
METTL3‐F	GGGTTGCACATTGTGTGGTC
METTL3‐R	GGCCTCCAAAGCTTCCACAT
HMBOX1‐F	CGCGTCTGTGTGTGTCTACT
HMBOX1‐R	TGCCAACTGCATTCTTCCCT
GAPDH‐F	CTTGTGCAGTGCCAGCCTC
GAPDH‐R	ACCAGCTTCCCATTCTCAGC

Abbreviations: F, forward primer; R, reverse primer.

### 2.9 Western blotting

Total protein was quantified using a BCA kit (23227, Thermo Fisher Scientific, USA). Protein samples were diluted in 5× sample buffer and then electrophoresed. Membranes loaded with separated proteins were first soaked in blocking solution for 60 min at room temperature and then incubated overnight at 4°C with primary antibodies (anti‐METTL3, 1:1000, ab195352, Abcam; anti‐HMBOX1, 1:1500, PA5‐21558, Invitrogen; anti‐GAPDH, 1:5000, ab6046, Abcam). After washing, the membranes were incubated with secondary antibodies (1:5000, ab150077, Abcam) at room temperature for 1 h. Blot images were captured using a BioSpectrum imaging system (UVP, USA).

### 2.10 Statistical analysis

GraphPad Prism 8.0 was used for statistical analysis. All quantitative data are presented as mean ± SEM. The Shapiro–Wilk test was performed to assess the normality of data. Parameters of more than two groups were compared using a one‐way analysis of variance followed by the Bonferroni test, while differences between two groups were analyzed using Student's *t*‐test.

## 3 RESULTS

### 3.1 CircCD44 promotes ESCC cell proliferation and invasion

**FIGURE 1:**
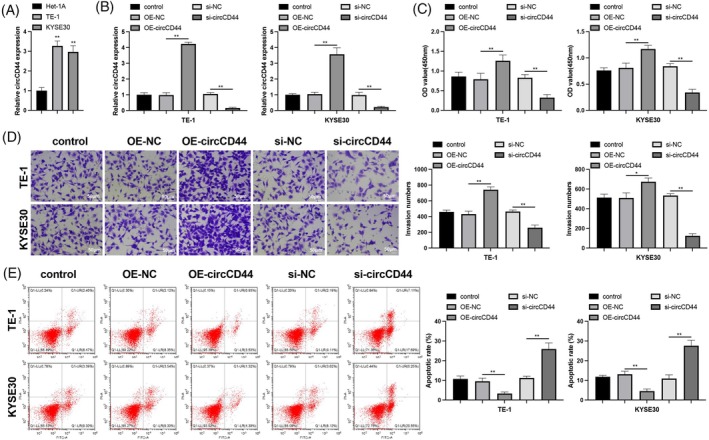
CircCD44 knockdown inhibits esophageal squamous cell carcinoma (ESCC) cell proliferation and invasion. ESCC cells were transfected with circCD44‐related vectors: (A, B) RT‐qPCR detection of circCD44; CCK‐8 (C), transwell (D), and flow cytometry (E) assessments of cell proliferation, invasion, and apoptosis. *N* = 3; *p < 0.05, **p < 0.01.

CircCD44 is involved in cancer progression, but its role in ESCC has been inadequately studied. An increase in circCD44 expression was detected in ESCC cell lines (Figure 1A, *p* < 0.05). To investigate the influence of circCD44 on ESCC cells, we transfected circCD44‐related vectors into ESCC cells and then measured phenotypic changes. RT‐qPCR results confirmed successful transfection; circCD44 expression was upregulated following OE‐circCD44 transfection and downregulated following si‐circCD44 transfection (Figure 1B, *p* < 0.05 vs. OE‐NC and si‐NC, respectively). Phenotypic analysis showed that circCD44 overexpression enhanced the proliferative and invasive abilities of ESCC cells while reducing their apoptosis; conversely, the opposite results were observed when circCD44 was silenced (Figure 1C–E, *p* < 0.05). These findings indicate that circCD44 promotes ESCC cell proliferation, invasion, and resistance to apoptosis in vitro.

### 3.2 HMBOX1 stimulates ESCC cell proliferation and invasion

**FIGURE 2:**
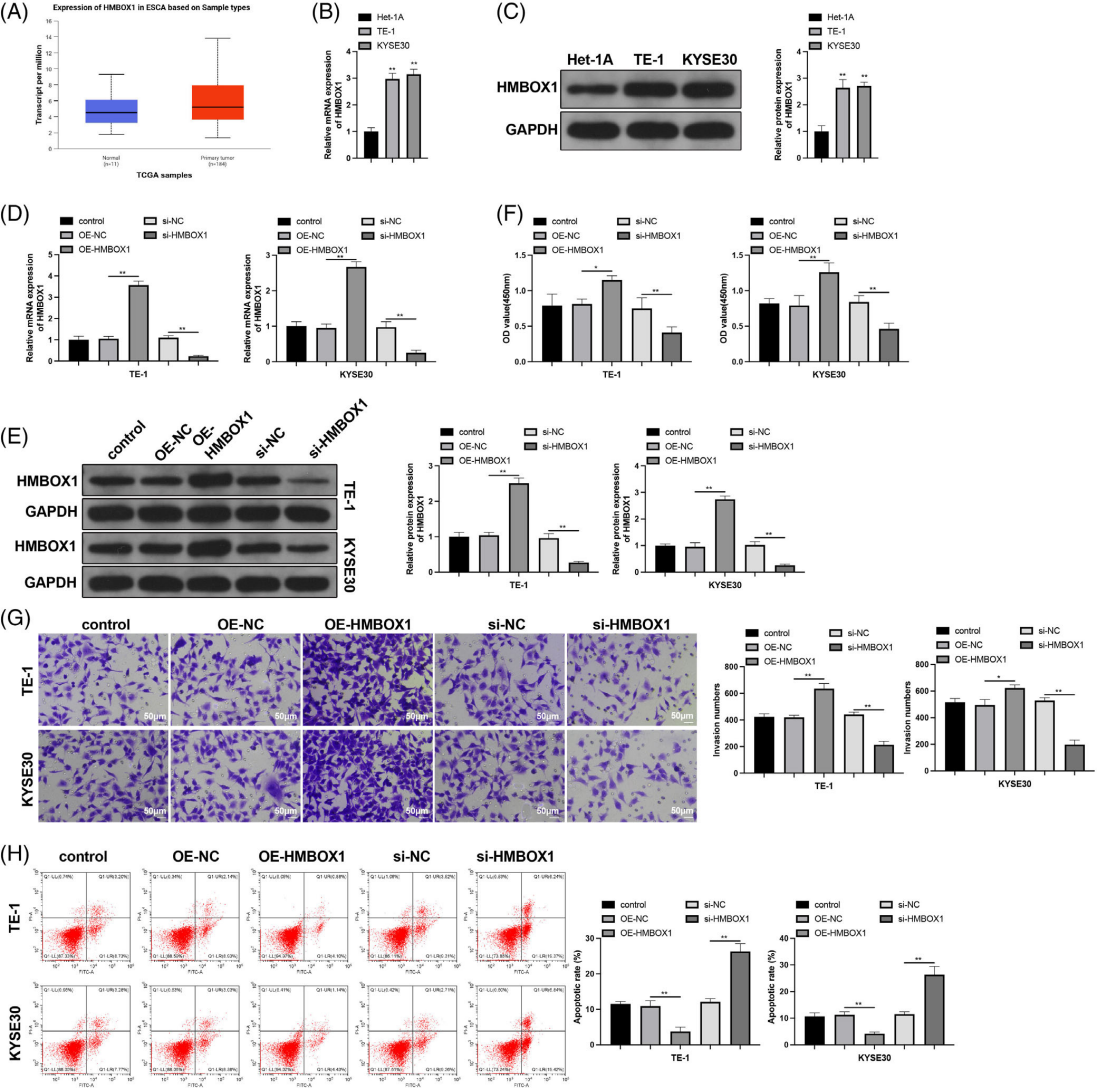
Homeobox containing 1 (HMBOX1) knockdown suppresses esophageal squamous cell carcinoma (ESCC) cell proliferation and invasion. ESCC cells were transfected with HMBOX1‐related vectors: (A) HMBOX1 expression in patients with ESCC predicted by the TCGA database (https://ualcan.path.uab.edu/); RT‐qPCR (B, D) and western blot (C, E) detection of HMBOX1 mRNA and protein; CCK‐8 (F), transwell (G), and flow cytometry (H) assessments of cell proliferation, invasion, and apoptosis. *N* = 3; *p < 0.05, **p < 0.01.

HMBOX1 has been identified as a potential biomarker for various cancers. The TCGA database showed that HMBOX1 is highly expressed in patients with ESCC (Figure 2A). HMBOX1 expression levels were also found to be higher in ESCC cell lines (Figure 2B,C, *p* < 0.05). ESCC cells were transfected with OE‐HMBOX1 and si‐HMBOX1, which successfully upregulated and downregulated HMBOX1 expression, respectively, as demonstrated by the PCR and western blot data (Figure 2D,E, *p* < 0.05 vs. OE‐NC and si‐NC, respectively). Phenotypic analysis revealed that HMBOX1 overexpression enhanced the proliferative and invasive abilities of ESCC cells while reducing apoptosis; HMBOX1 silencing elicited the opposite results (Figure 2F–H, *p* < 0.05). Collectively, these results demonstrate an oncogenic role of HMBOX1 in ESCC cell lines.

### 3.3 CircCD44 regulates HMBOX1 through METTL3

**FIGURE 3:**
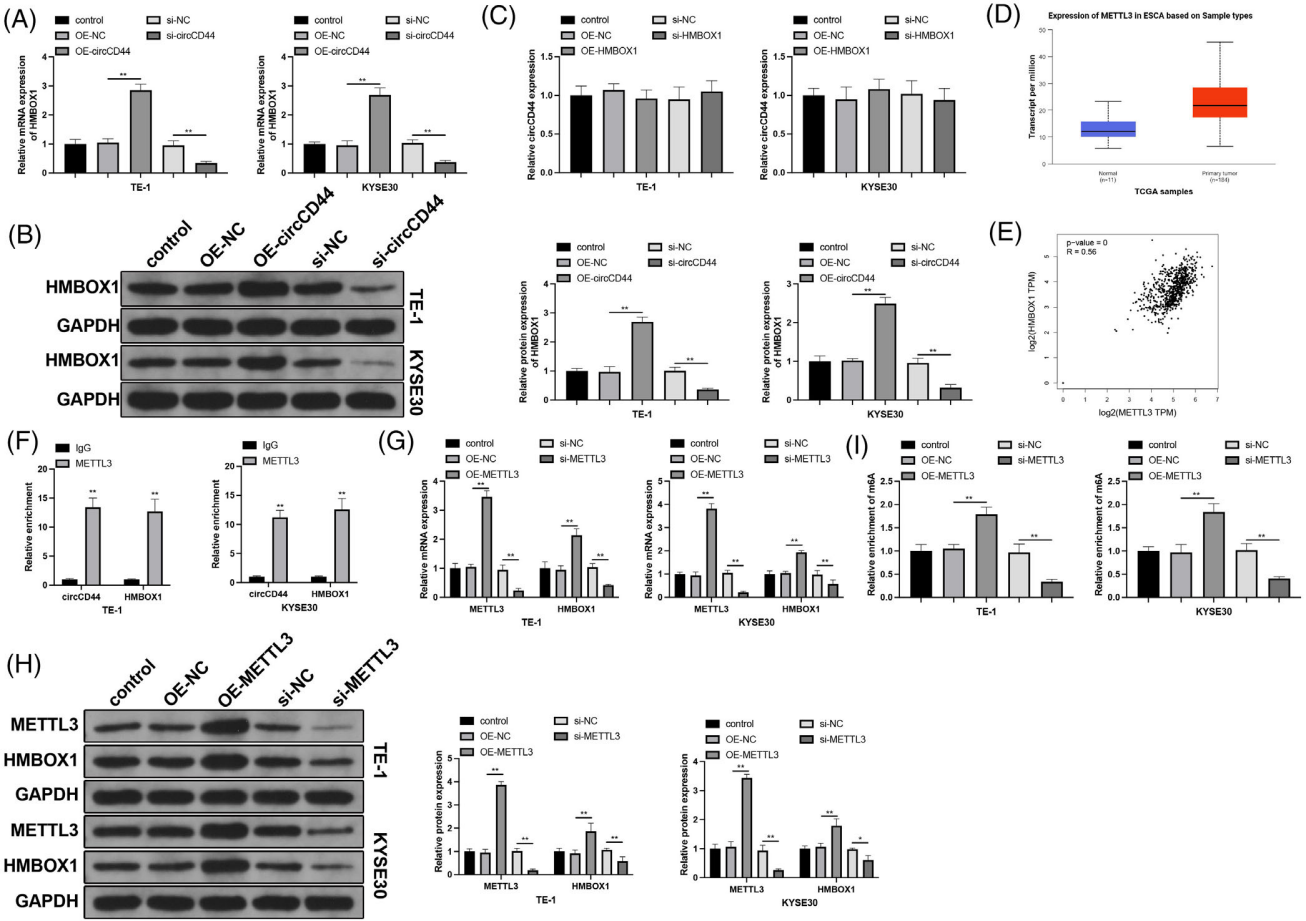
CircCD44 modulates homeobox containing 1 (HMBOX1) expression through METTL3. The mechanism of circCD44 regulating HMBOX1 was investigated: RT‐qPCR (A, C) and western blot (B) detection of circCD44 and HMBOX1 expression; (D) METTL3 expression in patients with esophageal squamous cell carcinoma (ESCC) predicted by the TCGA database; (E) GEPIA database (http://gepia.cancer‐pku.cn/) analysis of the correlation between METTL3 and HMBOX1 expression in ESCC; (F) RNA immunoprecipitation (RIP) detection of METTL3 binding to circCD44 and HMBOX1 mRNA; RT‐qPCR (G) and western blot (H) detection of METTL3 and HMBOX1 expression; (I) Me‐RIP detection of HMBOX1 mRNA m^6^A modification. *N* = 3; *p < 0.05, **p < 0.01.

Next, we investigated the regulatory effects of circCD44 and HMBOX1 on each other's expression. CircCD44 overexpression promoted HMBOX1 expression, while circCD44 inhibition reduced HMBOX1 expression (Figure 3A,B, *p* < 0.05). However, altering HMBOX1 expression did not affect circCD44 expression (Figure 3C, *p* > 0.05). Previous studies have shown the involvement of the m^6^A methyltransferase METTL3 in ESCC progression.^19^, ^20^ The TCGA database indicated that METTL3 is highly expressed in ESCC (Figure 3D). The starBase database showed that METTL3 has binding sites in circCD44 and HMBOX1 mRNA. Additionally, the GEPIA database showed a positive correlation between METTL3 and HMBOX1 in ESCC (Figure 3E). Therefore, we speculated that circCD44 regulates HMBOX1 mRNA through METTL3. The RIP assay in ESCC cells showed enrichment of circCD44 and HMBOX1 mRNA in the METTL3 pull‐down products, indicating binding of METTL3 to circCD44 and HMBOX1 mRNA (Figure 3F, *p* < 0.05). Transfection‐induced METTL3 overexpression and knockdown respectively increased and decreased HMBOX1 expression (Figure 3G,H, *p* < 0.05). Additionally, METTL3 did not affect circCD44 expression. Me‐RIP assay results showed that the m^6^A modification level of HMBOX1 mRNA was elevated by METTL3 overexpression and reduced by METTL3 knockdown (Figure 3I, *p* < 0.05). Altogether, these findings suggest that circCD44 promotes m^6^A modification of HMBOX1 mRNA by binding to METTL3, thereby upregulating HMBOX1 expression.

### 3.4 CircCD44 regulates METTL3/HMBOX1 to facilitate ESCC cell proliferation and invasion

**FIGURE 4:**
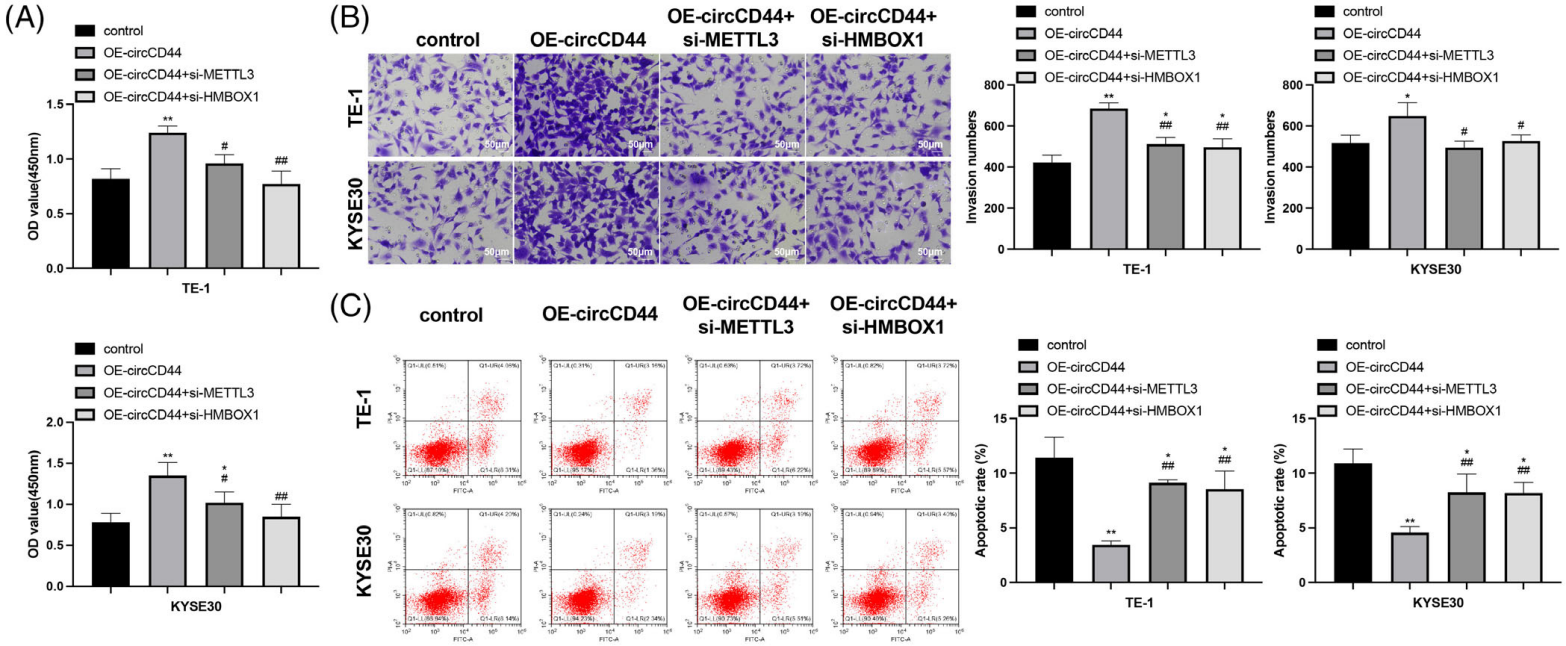
CircCD44 regulates METTL3/homeobox containing 1 (HMBOX1) to facilitate esophageal squamous cell carcinoma (ESCC) cell proliferation and invasion. ESCC cells were transfected with OE‐circCD44, OE‐circCD44 + si‐METTL3, or OE‐circCD44 + si‐HMBOX1: CCK‐8 (A), transwell (B), and flow cytometry (C) assessments of cell proliferation, invasion, and apoptosis. *N* = 3; *p < 0.05 and **p < 0.01, compared to control; ^#^ *p* < 0.05 and ^##^ *p* < 0.01, compared to OE‐circCD44.

To confirm whether circCD44 targets the METTL3/HMBOX1 axis to promote ESCC progression in vitro, we examined phenotypic changes of ESCC cells transfected with OE‐circCD44, OE‐circCD44 + si‐METTL3, or OE‐circCD44 + si‐HMBOX1. METTL3 or HMBOX1 knockdown suppressed proliferation and invasion and stimulated apoptosis in ESCC cells overexpressing circCD44 (Figure 4A–C, *P* < 0.05). This indicates that circCD44 exerts its oncogenic effect in ESCC cell lines by targeting the METTL3/HMBOX1 axis.

### 3.5 CircCD44 promotes ESCC growth in vivo through the METTL3/HMBOX1 axis

**FIGURE 5:**
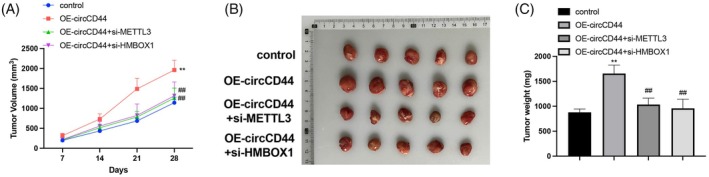
CircCD44 targets METTL3/homeobox containing 1 (HMBOX1) to promote esophageal squamous cell carcinoma growth. TE‐1 cells were injected subcutaneously into nude mice to establish a xenograft model: (A) Tumor growth was monitored; (B) Tumors were isolated and observed; (C) Tumor weight was measured. *N* = 5; **p < 0.01, compared to control; ^##^ *p* < 0.01, compared to OE‐circCD44.

TE‐1 cells transfected with OE‐circCD44, OE‐circCD44 + si‐METTL3, or OE‐circCD44 + si‐HMBOX1 were subcutaneously transplanted into nude mice to observe the regulatory effect of circCD44 on ESCC growth. CircCD44 overexpression accelerated tumor growth and increased tumor size (Figure 5, *p* < 0.05). METTL3 or HMBOX1 knockdown blunted the oncogenic effect of circCD44 overexpression in vivo.

## 4 DISCUSSION

ESCC is a highly heterogeneous disease with poor prognoses. In recent years, circRNAs have been extensively implicated in various cancers, including ESCC, showing great promise as prognostic biomarkers and therapeutic targets. Aiming to explore the circRNA landscape of ESCC, this study focused on an ESCC‐associated circRNA, circCD44, and uncovered a novel mechanism of action. Specifically, circCD44 binds to METTL3 to catalyze m^6^A modification in HMBOX1 mRNA, thereby promoting both HMBOX1 mRNA and protein expression. Further rescue experiments in ESCC cell lines and nude mice validated that circCD44 promotes ESCC progression by regulating the METTL3/HMBOX1 axis.

CircCD44 is a newly identified circRNA with its function largely unexplored. Meng et al. reported that circCD44 is highly expressed in patients with ESCC and promotes proliferation, invasion, and migration of ESCC cells both in vitro and in vivo.^13^ Consistent with these findings, the present study detected upregulated expression of circCD44 in ESCC cell lines TE‐1 and KYSE30 and found that circCD44 promoted the proliferation and invasion of ESCC cells in vitro and the growth of TE‐1‐derived tumors in nude mice. Additionally, circCD44 suppressed ESCC cell apoptosis. Apart from ESCC, circCD44 also plays a role in other cancers. Reduced LRRC4‐SAM68 interaction can downregulate circCD44 expression and subsequently affect the miR‐326/miR‐330‐5p/SMAD6 axis downstream of circCD44 in glioblastoma, influencing its progression.^11^ Moreover, circCD44 expression correlates with poor prognosis in triple‐negative breast cancer by sponging miR‐502‐5p and interacting with IGF2BP2.^10^ Furthermore, circCD44 promotes oxaliplatin resistance in colorectal cancer cells by sponging miR‐330‐5p to upregulate ABCC1 expression.^12^ These findings demonstrate the significant impact of circCD44 dysregulation on cancer progression through various molecular pathways. Unlike the previously identified miR‐based mechanisms,^13^ this study revealed a role for circCD44 in ESCC progression through the RBP METTL3.

METTL3 is a core component of the m^6^A methyltransferase complex, where it plays a catalytic role.^21^ m^6^A is one of the most common chemical modifications in eukaryotic RNAs, modulating RNA stability, translation, splicing, and transport.^22^ METTL3‐dependent m^6^A methylation has been reported to positively regulate ESCC malignancy by affecting c‐Myc and IFIT2.^17^, ^23^ In this study, we identified a novel target of METTL3 in ESCC cells, HMBOX1, which was previously demonstrated to be stabilized by METTL3‐mediated m^6^A modification.^24^ Consistent with this finding, METTL3 promoted m^6^A modification of HMBOX1 mRNA and enhanced HMBOX1 expression in this study.

HMBOX1 is a transcription factor widely expressed in normal human tissues and is involved in embryonic development, differentiation, inflammation, tumorigenesis, and telomerase regulation.^25^ The tumor‐promoting effects of HMBOX1 have been analyzed in various cancers, such as osteosarcoma, cervical cancer, and lung squamous cell carcinoma.^15^, ^26^, ^27^ However, its role in ESCC remains unclear. This study is the first to demonstrate that HMBOX1 promotes ESCC cell proliferation, invasion, and apoptosis resistance in vitro and accelerates the growth of TE‐1‐derived tumors in nude mice. Previously, METTL3‐catalyzed m^6^A in HMBOX1 mRNA was shown to induce genomic instability in cancers by disrupting telomere homeostasis.^16^ The interplay between METTL3, m^6^A, HMBOX1, and telomeres in ESCC was not explored in this study and warrants further investigation.

In summary, circCD44 promotes HMBOX1 expression by binding to METTL3 to upregulate HMBOX1 mRNA m^6^A modification, thereby promoting ESCC progression. This study reveals a novel mechanism of circCD44 in ESCC, which may guide the development of targeted therapies for ESCC. However, the downstream signaling pathways of HMBOX1 in ESCC have not yet to be determined, and will be an important direction for subsequent research.

## CONFLICT OF INTEREST STATEMENT

The authors declare no conflict of interest.

## ETHICS STATEMENT

All experimental procedures were approved by the Institutional Animal Care and Use Committee (IACUC) of Longgang Center Hospital of Shenzhen.

## Data Availability

The datasets used or analyzed during the current study are available from the corresponding author on reasonable request.
